# UK publicly funded Clinical Trials Units supported a controlled access approach to share individual participant data but highlighted concerns

**DOI:** 10.1016/j.jclinepi.2015.07.002

**Published:** 2016-02

**Authors:** Carolyn Hopkins, Matthew Sydes, Gordon Murray, Kerry Woolfall, Mike Clarke, Paula Williamson, Catrin Tudur Smith

**Affiliations:** a MRC North West Hub for Trials Methodology Research, Department of Biostatistics, University of Liverpool, Block F Waterhouse Building, 1-5 Brownlow Street, Liverpool, L69 3GL, UK; b MRC Clinical Trials Unit, University College London, Aviation House, 125 Kingsway, London, WC2B 6NH, UK; c Centre for Population Health Sciences, University of Edinburgh, Teviot Place, Edinburgh, EH8 9AG, UK; d MRC North West Hub for Trials Methodology Research, Department of Psychological Sciences, Block B Waterhouse Building, Brownlow Street, Liverpool L69 3GL, UK; e All-Ireland Hub for Trials Methodology Research, School of Medicine, Dentistry and Biomedical Sciences, Queen's University Belfast, Health Sciences Building, 97 Lisburn Road, Belfast, BT9 7BL, UK

**Keywords:** Data sharing, Individual participant data, IPD, Clinical trial, Publicly funded, Clinical trial unit

## Abstract

**Objectives:**

Evaluate current data sharing activities of UK publicly funded Clinical Trial Units (CTUs) and identify good practices and barriers.

**Study Design and Setting:**

Web-based survey of Directors of 45 UK Clinical Research Collaboration (UKCRC)–registered CTUs.

**Results:**

Twenty-three (51%) CTUs responded: Five (22%) of these had an established data sharing policy and eight (35%) specifically requested consent to use patient data beyond the scope of the original trial. Fifteen (65%) CTUs had received requests for data, and seven (30%) had made external requests for data in the previous 12 months. CTUs supported the need for increased data sharing activities although concerns were raised about patient identification, misuse of data, and financial burden. Custodianship of clinical trial data and requirements for a CTU to align its policy to their parent institutes were also raised. No CTUs supported the use of an open access model for data sharing.

**Conclusion:**

There is support within the publicly funded UKCRC-registered CTUs for data sharing, but many perceived barriers remain. CTUs are currently using a variety of approaches and procedures for sharing data. This survey has informed further work, including development of guidance for publicly funded CTUs, to promote good practice and facilitate data sharing.

## 1 Introduction

**What is new?**

• Publicly funded Clinical Trial Units (CTUs) have a crucial role to play in the sharing of individual participant level data from clinical trials.
• There is a lack of information available on current data sharing practice, level of support, and barriers to data sharing within publicly funded CTUs.
• This study has demonstrated that there is general support for clinical trial data sharing among publicly funded CTUs.
• However, there are concerns amongst the community about the potential misuse of data and the resource implications of data sharing activities.
• Several publicly funded CTUs are already involved with data sharing, but further guidance is now needed to help increase this and identify good practice.

Historically, many researchers have considered data generated in the conduct of a clinical trial as “private property” belonging to the trial sponsor or original research group [1]. As a result, further use of the data has often been restricted to those researchers, possibly limiting the research potential of valuable data. Progress is being made, and the potential added value of sharing clinical trial data is becoming more widely accepted. In particular, the sharing of patient-level data could enhance many research-related activities [2], [3]. Secondary analyses and meta-analysis of individual participant data (IPD) could reveal directions for future research and reduce the requirement for further clinical trials, and therapies could be made available to patients more quickly [4], [5].

The incentives for sharing clinical trial data in conjunction with concerns of publication bias and selective reporting practices have led to significant growth in support for initiatives that could lead to greater trial transparency, aiming to promote open science, benefit the public's health, and reduce wasteful research [6], [7], [8], [9], [10]. Recently, focus on sharing data from clinical trials has led to consideration of how industry, regulatory bodies, and clinical trial funders can amend their practices to facilitate clinical trial transparency. The AllTrials campaign [11] calls for all past and present clinical trials to be registered and their full methods and summary results reported and has the support of representatives from regulatory bodies, industry, publishing groups, research funders, and many others. The European Medicines Agency (EMA) has recently released a policy for “Publication and access to clinical trial data” [12], and the Association of the British Pharmaceutical Industry, the Institute of Medicine, and the European Forum for Good Clinical Practice are among many groups to have held workshops discussing sharing clinical trial data and ensuring transparency [13], [14], [15]. The British Medical Journal (BMJ) strongly supports transparency and data sharing with a policy stating that “from January 2013 trials of drugs and medical devices will be considered for publication only if the authors commit to making the relevant anonymized patient-level data available on reasonable request” [16]. Significant steps toward transparency are being made by some pharmaceutical companies. The collaborative Web site https://www.clinicalstudydatarequest.com/ provides a route to request access to anonymized patient-level data and supporting documents from multiple sponsors [17], [18]. Separately, the Johnson & Johnson agreement with the Yale University Open Data Access (YODA) project allows third party access to their data [19].

Much of the publicity surrounding the topic of clinical trial data transparency has featured stakeholders involved with commercially funded clinical trials. However, clinical trials are also designed, coordinated, analyzed, and reported by publicly funded sponsors. Indeed, 58% of intervention trials registered in clinicaltrials.gov (October 2014) have nonindustry sponsors, and they too have a duty to consider procedures to make patient-level data available. Rathi et al. [20] surveyed 317 trialists from a range of sectors to evaluate levels of support and concerns associated with clinical trial data sharing, and the Cochrane IPD Meta-analysis Methods Group was surveyed in 2011 [21]. Both surveys demonstrated that public sector researchers are generally in support of making clinical trial data available. This is further evidenced by several examples of publicly funded clinical trialists making data available through open access systems (e.g., Virtual International Stroke Trials Archive [VISTA], Alzheimer’s Disease Neuroimaging Initiative [ADNI], and The National Institute of Diabetes and Digestive and Kidney Diseases [NIDDK]) [22], [23], [24], by providing IPD for meta-analysis (e.g., ACCENT database, INDANA) [25], [26] and as part of wider genetic data consortia (e.g., Biomarkers Consortium, PRO-ACT) [27], [28]. Despite this progress, accessing clinical trial data from publicly funded clinical trials can sometimes be difficult, if not impossible, and further steps are needed to encourage and facilitate future sharing.

The UK Clinical Research Collaboration (UKCRC)–registered Clinical Trial Unit (CTU) network includes 45 publicly funded CTUs that design, conduct, analyze and publish clinical trials across different diseases and settings. The CTUs are notable in that they can be involved in both sides of data sharing; requests for data are made to the units from external researchers, and members within the units can also be involved in requesting data from other sources. The CTUs are a potential vehicle to facilitate data sharing of publicly funded trials in the United Kingdom and could provide a model of good practice for other publicly funded trials. Therefore, we surveyed CTU directors to capture current practice, identify perceived barriers, and explore attitudes to help inform the development of guidance to facilitate data sharing of publicly funded trials.

## 2 Methods

### 2.1 Survey instrument development

We developed a 47-item questionnaire as part of an MRC-funded project. Questions were selected by the research team using their expertise and experiences of data sharing activities. The survey was developed and conducted online using SelectSurvey.NET. The complete survey is provided as Supplementary Material/Appendix at www.jclinepi.com. A brief synopsis and link to the survey was e-mailed to the UKCRC-registered CTU Directors in April 2014. E-mail reminders were sent after 2, 4, and 6 weeks. Multiple responses from the same unit were combined where complementary; otherwise, the response of the most senior CTU member was used.

The questionnaire took approximately 15 minutes to complete. Ethical approval was obtained from the University of Liverpool Research Ethics Committee. As the survey was conducted online, completion was regarded as consent to participate.

### 2.2 Survey domains

Multiple choice, Likert scale, and free-text questions covered the areas of:

1. Current practice
2. Custodian experience
3. Requester experience
4. Future perspective
5. Standards and awareness
6. Models for data sharing
7. Requirements and potential problems

### 2.3 Response analysis

Free-text responses were reviewed and categorized by two of the research team (C.H. and C.T.S.) with categorizations compared and agreed. Results were summarized using descriptive statistics.

## 3 Results

There were 24 responses from 23 (51%) registered CTUs across the United Kingdom (two responses were received from the same CTU). Three of the responders provided partial information and have therefore only been included in relevant sections of the results. From 24 responses, the majority (71%) were completed by the CTU Director or Deputy Director with seven (29%) completed by delegated members of the CTUs including statisticians, operations managers, data managers, and trial managers. Responding CTUs conducted trials in many diseases, all trial phases, and included clinical trials of investigational medicinal products (CTIMPs) and non-CTIMP trials; there were no specific trial phases, disease types, or methodological research areas that were underrepresented in the survey compared with nonresponding CTUs.

### 3.1 Current practice

Five of the 23 CTUs (22%) had an established Data Sharing Policy, 11 (48%) had a policy in development, but seven (30%) had no immediate intention of developing a policy.

**Fig. 1:**
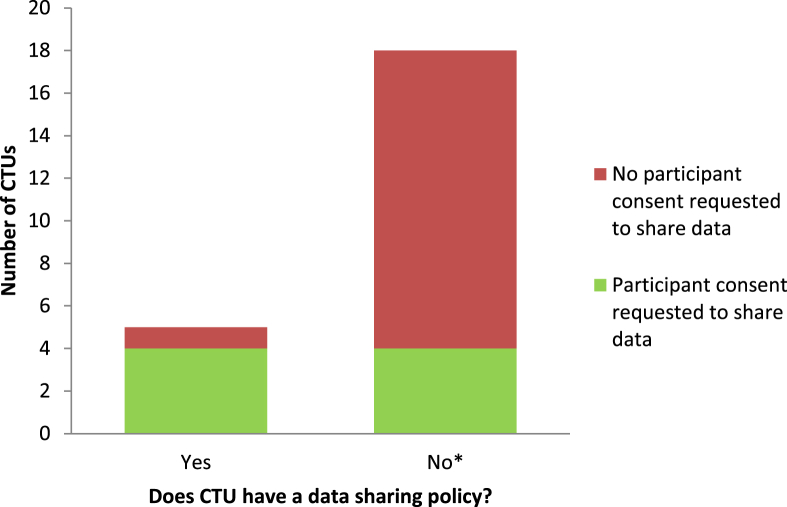
Current practice regarding data sharing policy and consent from participants to share their data. *No policy includes seven CTUs with no immediate intention to develop a policy and 11 CTUs with a policy in development. CTUs, Clinical Trial Units.

Eight (35%) CTUs indicated that consent is generally sought from participants in clinical trials for their data to be used outside the original scope of trials (Fig. 1). However, a standard phrase was not being used across the CTUs. Examples of text used include: “agree to allow any information or results arising from this study to be used for health care and/or further medical research on the understanding that my identity will remain anonymous wherever possible”; consent for “medical data to be collected for this study and may be used to develop new research and that data protection regulations will be observed”; “used for future research and to be transferred to research institutes within the UK”; consent for separate blood samples to be “anonymized and stored for infection and immunity-related research in the future”; consent for “participants to be contacted independently by other researchers to take part in research within the same disease area.”

Of the 15 (65%) CTUs that do not currently specifically request consent for patient data to be used beyond the scope of the original trial, 12 (80%) would be prepared to request broader consent in the future (one only if the CTU signed up to a central repository in future), but two (13%) would not, giving the following reasons; “This should not be CTU policy—needs discussion and approval of other parties” and “This should be a condition of ethical approval or funding—the CTU only handles data on a subset of studies and this is a much wider issue.” One CTU did not provide a response.

### 3.2 CTU experience of sharing data

**Fig. 2:**
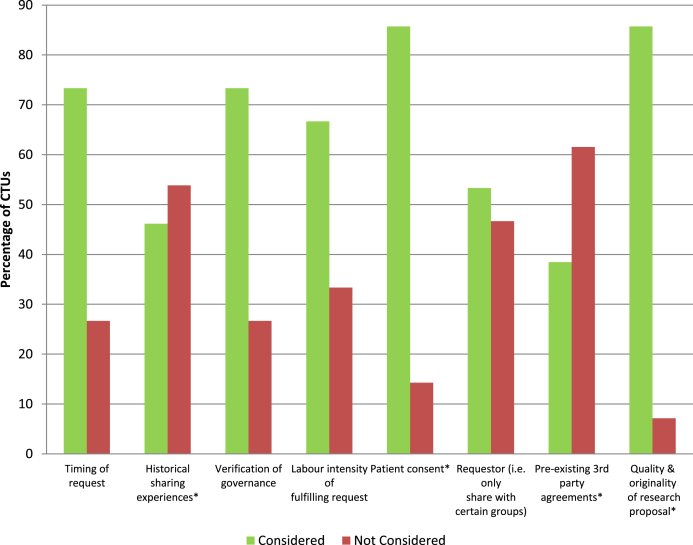
Considerations when reviewing requests for data as reported by 15 CTUs that had received a request in the past 12 months. *Total number of responses <15 as at least one CTU did not respond. CTUs, Clinical Trial Units.

Fifteen (65%) CTUs had received at least one request for data in the previous 12 months. Most commonly, two requests had been received, but four CTUs had received at least five data requests. Institutes of higher education were the most common requester of data (*n* = 10), followed by NHS Trust/Clinician (*n* = 6), independent researcher (*n* = 5), and industry (*n* = 4). The most common reason for requesting data was for meta-analysis. No recent requests had been refused, although some were only partially fulfilled due to conflict with other research projects or preplanned analyses within the CTU. Patient consent, quality and originality of research proposal, verification of governance, timing of request, and labor intensity of fulfilling the request were the most common considerations (reported by at least two-thirds of the CTUs) when reviewing requests for data from the CTU (Fig. 2). There were no reported issues with providing the data in the format that had been requested.

Fourteen (93%) of the 15 CTUs that received requests had also received a research proposal outlining the purpose of the data request for at least one request, but only nine (60%) CTUs used a data sharing agreement. Six (40%) CTUs received “Requester credentials to signify competence to analyze data,” and five (33%) had received “assurances that there would be no attempts to retrospectively identify patients” as part of their data sharing process.

CTUs that had been involved with sharing data were asked about the resources required and whether certain activities were more difficult for older trials (>5 years old) compared with more recent trials. The majority (93%) stated that the location and preparation of the data were resource intensive (defined in our survey as more than half a day to complete) and this was the most frequently cited activity (*n* = 6, 40%) felt to be more difficult for older trials compared with more recently completed trials. Ten (67%) CTUs described the anonymization of data as “not likely to be resource intensive,” and only two (13%) felt that this would be more difficult for older trials. Reviewing requests for validity was considered more difficult by three (20%) CTUs, and two (13%) described generating data sharing agreements as more difficult for historical data.

### 3.3 CTU experiences of requesting external data

Of all 23 responding CTUs, seven (30%) had made an external request for data in the last 12 months. Six of these provided additional information about the external requests; four CTUs (67%) had made two requests, and two CTUs (33%) had made more than five requests. These requests had been made to: “Institute of higher education” (*n* = 3), “Industry” (*n* = 2), “NHS Trust/Clinician” (*n* = 2), “Independent researcher” (*n* = 2), and “Other” [“NHS Data—Information Centre” (*n* = 1) and “Health and Social Care Information Centre” (*n* = 1)]. All six CTUs had made successful requests, and data had been provided either completely (*n* = 4, 67%) or partially (*n* = 2, 33%). There was considerable variation in the time taken between their initial request and the provision of data ranging from a few weeks to over a year. Reasons given for making the data requests were provided by the six CTUs as: meta-analysis (*n* = 4), follow-up of trial participants (*n* = 2), methodological research (*n* = 1), and feasibility of setting up registry (*n* = 1). All six CTUs had been required to submit a research proposal and data sharing agreement, and some were required to provide assurances they would not attempt to retrospectively identify patients (*n* = 3) and provide credentials or proof of competency to analyze the data being requested (*n* = 2).

### 3.4 Future perspectives

#### 3.4.1 Standards and awareness

**Fig. 3:**
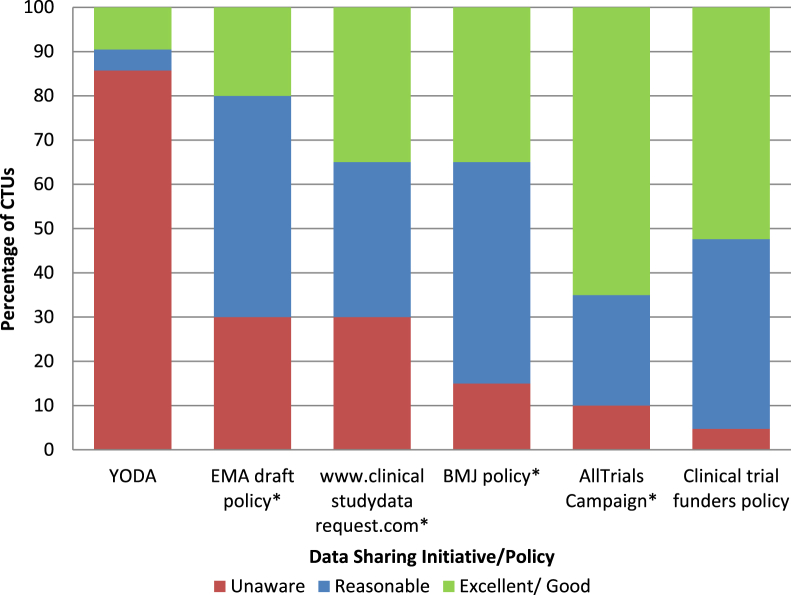
Awareness of various data sharing initiatives and policies. *Total response is <21 because CTUs failed to provide a response or responded N/A. YODA, Yale University Open Data Access; EMA, European Medicines Agency; BMJ, British Medical Journal; CTUs, Clinical Trial Units.

Twenty-one CTUs rated their knowledge of various data sharing initiatives, policies, and incentives. The AllTrials campaign was the most well known, with 13 (65%) responders indicating they had “excellent” or “good” knowledge of the campaign (one nonresponse). The YODA initiative was least well known with 18 (86%) “unaware” of the project. Responders had some awareness of the draft EMA Policy, clinicalstudydatarequest.com Web site, the BMJ policy on data sharing, and data sharing policies of clinical trial funders (Fig. 3). Examples of other initiatives or policies that respondents were aware of included the “new EU Clinical Trial regulation,” “NHS Information Governance and the NHS Consortium,” “US Institute of Medicine,” “PLOS,” and “NIH” policies.

As the use of common data standards across trials could simplify data sharing, the CTUs were asked if there were any data standards commonly used within the unit. Eleven of 21 (52%) CTUs stated that they apply standard formats for their electronic data. MedDRA (Medical Dictionary for Regulatory Activities) was the most frequently named standard (*n* = 6), but CDISC (Clinical Data Interchange Standards Consortium) (*n* = 2) and CTCAE (Common Terminology Criteria for Adverse Events) v4.0 (*n* = 1) were also mentioned.

Twelve of 21 CTUs (57%) would be prepared to adopt a standard data sharing policy (standardized for the UKCRC CTUs as a minimum), but nine (43%) indicated that there were specific reasons or external influences that would prevent their CTU adopting a standard data sharing policy—these were categorized as: process cannot be standardized; standardizing data too complicated; overarching university and NHS policy on data sharing; variety of governance structures for different data types; conditions to involve the trial team in review of data request and include trial investigator on publication; burden on original researchers; patent/IP issues; ownership of data; misuse/incorrect secondary analysis; participant consent; ethical approval; logistical; and cost implications. Fourteen of 20 (70%) CTUs would be prepared, in principle, to transfer data to a central repository assuming it was legal and ethical. Many used a free-text box to indicate associated concerns about confidentiality, funding, and the issue of data ownership.

#### 3.4.2 Models for data sharing

**Fig. 4:**
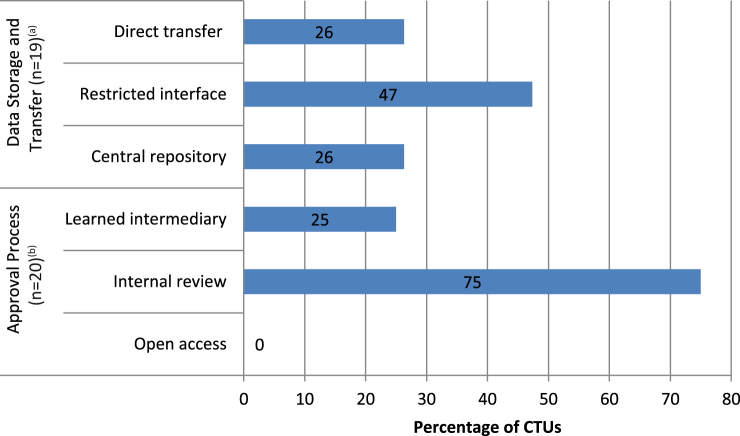
Models rated “Most suitable” by responding CTUs. ^a^Data storage and transfer. Direct transfer: data transferred to external users for secondary analyses. Restricted interface: data remain with the data custodian, but access is granted for external users to analyze data through a specific interface. Central repository: data are uploaded to, and downloaded from, a central independent repository. ^b^Approval process. Learned intermediary: an Independent Review Board reviews requests and judges them based on criteria such as science, benefit-risk analysis, and competence of the requestor to perform the specified analyses. Internal review: specific detailed requests are placed with the custodian who assesses the request based on science, benefit-risk analysis, and competence of the requestor to perform the specified analyses. Open access: no approval required, data available for any user to access. CTUs, Clinical Trial Units.

An “internal review” model, in which the data custodian would review and assess a request based on criteria such as scientific soundness of the proposal or competence of the requestor to perform the specific proposed analyses, was considered the most suitable model for granting access to data (*n* = 15 of 20, 75%). Five (25%) CTUs preferred a “learned intermediary” model with requests for data reviewed independently by a review panel. Notably, none of the CTUs considered an “open access” model with no required approval process as “most suitable” (Fig. 4). CTUs commonly considered (*n* = 9 of 19, 47%) access through “restricted interface” model as most suitable for data provision; the data custodian maintains possession but grants access to an external requestor through a specific secure interface with restrictions on data download. Five (26%) supported an approach whereby data would be uploaded to and downloaded from a central independent repository, and an equal number (*n* = 5, 26%) viewed “direct transfer” of data to external parties without a repository as most suitable (Fig. 4). One responder included a further suggestion for “controlled access with active engagement from trial team as research partners.”

Twenty-one CTUs provided opinion about the most appropriate time for making data available at the end of a trial; 10 (48%) CTUs selected “At any time after the trial team have completed all analyses and secondary exploratory analyses”; 1 (5%) selected “As soon as final analysis is complete”; 2 (10%) chose “within 12 months of last patient last visit”; 1 (5%) chose “within 24 months of last patient last visit”; 6 (29%) CTUs provided free-text responses categorized as: timing would vary by trial [2]; after the trial, results have been published [4]. One (5%) provided a data sharing policy which suggested a period of exclusivity determined on a per trial basis (generally a minimum of 5 years from last patient last visit).

#### 3.4.3 Potential problems

**Fig. 5:**
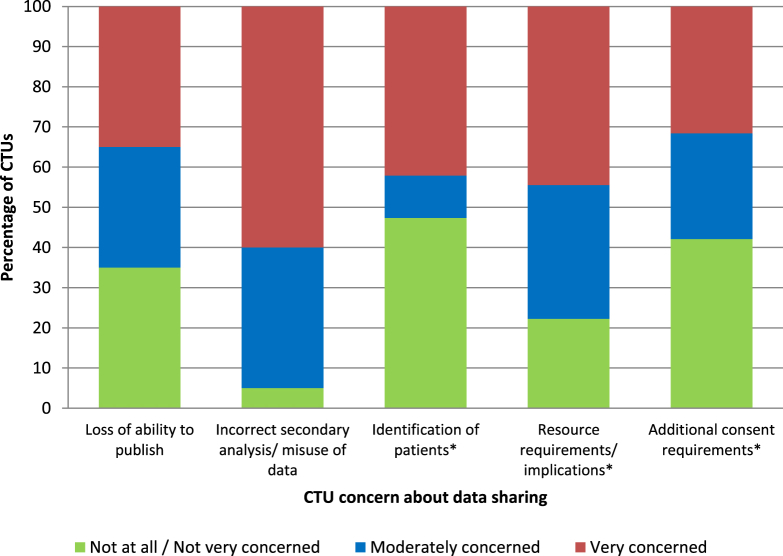
Level of concern about frequently raised barriers to making full clinical trial data available on a controlled access platform. *Total response was <20 because CTUs failed to provide a response or responded N/A. CTUs, Clinical Trial Units.

Twenty CTUs provided at least one response to a Likert scale question (Fig. 5) addressing levels of concern on specific topics. The risk of incorrect secondary analyses and misuse of data was of greatest concern with all but one CTU stating they were moderately (*n* = 7 of 20, 35%) or very concerned (*n* = 12 of 20, 60%), and most CTUs were moderately (*n* = 6 of 18, 33%) or very concerned (*n* = 8 of 18, 44%) about the resource implications of sharing data. CTUs were fairly evenly split across being very (*n* = 7 of 20, 35%), moderately (*n* = 6 of 20, 30%), or not very concerned (*n* = 7 of 20, 35%) about the loss of IP/ability to publish. The “Identification of patients” and “additional consent requirements” split the CTU opinion with approximately half being “very” or “moderately” concerned and the other half “not very” or “not at all” concerned. Some “Other” concerns were raised by three CTUs, specifically the qualifications of applicants to analyze data, repository security, patent protection, and potential impact on future trial recruitment.

CTUs were asked to indicate problems foreseen if all data (including IPD) were to be published on a controlled access platform. Eleven of 23 (48%) CTUs raised concerns about time and resource implications of making data sets available (*n* = 6), getting all CTUs on board with a central repository or standard data formats (*n* = 4), how to deal with subsets of patients withdrawing consent or not providing it in the first place (*n* = 3), the right of the sponsors or investigators to maintain rights over the use of the data and to be acknowledged appropriately where data are shared (*n* = 4), and the risks of “bad research” both to the original research group and patients (*n* = 3).

## 4 Discussion

This survey explores the data sharing views and experiences of publicly funded CTUs in the United Kingdom. The media attention that has surrounded the issue of clinical trial data sharing within the last few years has mostly focused on CTIMPs conducted within the pharmaceutical industry, but most of the issues, and the need to share data, apply equally to publicly funded clinical trials. The CTUs that responded to this survey are fundamentally different to the pharmaceutical industry. They are involved in a diverse range of trials including CTIMPs, device trials, surgical trials, pragmatic trials, and observational studies. They do not set out to make a profit from the sale of the interventions being investigated, and trials within their portfolio are typically funded by public or charitable organizations. This often creates complex sponsorship arrangements that may introduce a level of ambiguity about ownership and responsibilities for sharing the data generated in the clinical trial. Publicly funded CTUs may also be linked to, or be part of, parent organizations such as Universities or NHS Trusts, and they may also conduct clinical trials in collaboration with industry, hence, may need to consider these overarching data sharing requirements in addition to those of the CTU, the funder, potential journal, and any relevant regulatory requirements. We would therefore strongly recommend that the issue of data sharing is explicitly and thoroughly discussed between stakeholders during the planning of a publicly funded trial to agree roles and responsibilities. Indeed, we fully support the recommendation in the SPIRIT 2013 checklist that a dissemination policy should be described in the trial protocol, with specific reference to “Plans, if any, for granting public access to the full protocol, participant-level data set, and statistical code” [29].

Our survey showed a variety of attitudes toward data sharing and different levels of activity and knowledge about current data sharing initiatives. CTUs may not yet have developed their procedures for sharing data due to a low demand and inadequate resources. However, if CTUs were to take a more proactive role in sharing data, awareness of the wealth and potential value of the data that are available could be increased. Of course this would have a resource implication which is a common concern and potential barrier for publicly funded CTUs. Funds may be provided to a sponsor to facilitate data sharing in future trials, but historically, there has been no provision to cover costs of wider data dissemination. Trialists now need to plan ahead for this and include appropriate costs for data set preparation and sharing in the same way as may be done for open access publication or archiving trial data.

Consent from patients to share anonymized data is often viewed as a prerequisite for data sharing, and one simple approach to remove this perceived barrier for future trials would be to actively seek consent from patients for this purpose. Our survey suggests that only a minority of publicly funded CTUs currently request consent for sharing data in their trials, yet most CTUs were supportive of a move toward such an approach. Furthermore, it is worth mentioning that even if explicit consent to share anonymized data has not been requested from patients in ongoing or completed trials, this does not preclude the sharing of anonymized data; the Data Protection Act no longer applies to data from deceased participants or data anonymized such that the individual is no longer identifiable [30]. As an example of this, the International Stroke Trial Collaborative Group has made the data from their publicly funded clinical trial publically available [22]. They note that “Consent for publication of raw data was not obtained from participants” and because “the data set is fully anonymous,” they present the view that “publication of the data set clearly presents no material risk to confidentiality of study participants.”

Those CTUs that had been approached for data in the last 12 months had provided data in all instances, albeit using different, possibly ad hoc, approaches. Adoption of a standard procedure, or at least some common principles across the CTUs, would greatly facilitate data sharing. The concept of a clinical trial data repository that CTUs could transfer data to was met positively, although there were differing opinions about the format this repository should take demonstrating the difficulty of a “one size fits all” approach. Nevertheless, not one CTU felt that an open access approach would be appropriate, the risks of bad research resulting from poor secondary analyses or incorrect replication of primary analyses, and risks of patient identification, could be mitigated through a controlled access system with data requests supported by evidence of the research team's expertise and a research plan providing details of proposed analyses. This practice has already been adopted by some CTUs and requesting this information or having to provide it does not seem to present a barrier to sharing activities. Furthermore, ensuring that the data sets are fully annotated and accompanied by all associated trial documentation such as the protocol and blank Case Report Forms should further minimize the possibility of conflicting results arising from replicated analyses. If reanalysis of the same data did produce conflicting results, then this should firstly be discussed with the original team, and if discrepancies remain, this information should of course be made publicly available.

Sharing IPD from clinical trials is only one part of a wider move toward “open science” that promotes open access to journal articles, protocols, source code, and data. Some may criticize an approach of sharing IPD with researchers within a controlled system as not being “open” enough and may advocate data being openly available for all to access without restriction. The clinical trials community must ensure that all reasonable precautions are taken to protect the privacy and confidentiality of trial participants and this should always be the overriding consideration. Taking unnecessary risks could lead to more harm than good, and we support the use of a controlled access approach as discussed by Sydes et al. [31].

### 4.1 Limitations of study

This survey was limited to UKCRC-registered CTUs and may not necessarily be representative of all UK CTUs or organizations that conduct publicly funded trials. It was targeted at CTU Directors but was completed by delegates in some cases. As the survey addressed current practices within the CTUs, there should not have been any impact of the responses being provided by someone other than the Director. However, it is possible that responses represent a partly political view rather than the personal opinion of scientists. The survey was short and simple, but only 51% of invitees provided useable responses; this level of response is typical of Web-based surveys [20], [32]. Reminders were sent regularly by e-mail, but more responses might have been received had follow-up occurred via telephone or if the survey remained open for longer. We feel that the responses provide a good representation of the UK stakeholder group; there was no clear underrepresentation of any particular subset of CTUs as a wide spread of phases, trial types, disease areas, and unit sizes were represented among the responding CTUs. Open questions allowing free-text answers enabled responders to present additional issues, concerns, and suggestions that may have been shared by other responders had they been presented as options within the original survey. Our threshold for defining “resource–intensive” one half-day (i.e., 4 hours) was too low.

### 4.2 Future directions

The information gathered indicates that there is general support for clinical trial data sharing within these publicly funded CTUs, but there are several perceived barriers that may be preventing initiatives from moving forward. This information has been used to inform the development of good practice guidance for publicly funded CTUs to encourage future data sharing [33].

### 4.3 Conclusions

Sharing clinical trial data is at the forefront of many discussions by regulatory bodies, research funders, and the pharmaceutical industry with an aim to increase transparency and facilitate efficiency and advances in research. Publicly funded CTUs have an important role to play in this arena. The wealth of experience and knowledge within the units could help establish an infrastructure to facilitate data sharing and improve the use of publicly funded clinical trial data for the public good. Action is now needed to focus efforts toward facilitating further clinical trial data sharing and developing good practice for data sharing in publicly funded CTUs.

## Supplementary data

Supplementary Material/Appendix
