# Supplementary material for: UK publicly funded Clinical Trials Units supported a controlled access approach to share individual participant data but highlighted concerns
Source: J Clin Epidemiol. 2016 Feb;70:17–25. doi: 10.1016/j.jclinepi.2015.07.002 (PMC4742521; doi:10.1016/j.jclinepi.2015.07.002)
Supplement: Supplementary Material/Appendix [file mmc1.doc]

Supplemental Material (Online Only)


Supplementary material: Survey distributed to potential respondents.

Purpose Of Survey

This survey is being conducted by researchers from the MRC Hubs for Trials Methodology Research and UK CRC registered CTUs. We estimate that it will take approximately 15 minutes to complete. The survey aims to seek opinions and details of current practice related to data sharing from across the UK CRC registered CTUs. In the context of this survey, 'data' refers to the underlying individual participant data and accompanying essential documents (e.g. protocol, annotated case report forms) from a clinical trial. Results from
this survey will inform the development of practical guidance on data sharing and further input will be requested from the UK CRC CTUs at a later date.

1.	Name:
2.	Role/Job Title:
3.	Institute/Unit:

Current Practice

Please answer the following questions to establish a picture of the current practices utilised at your CTU.

4.	Does your unit have a data sharing policy?
o	No – there is no intention to develop a policy o	No – but there is a policy in development
o	Yes

5.	Does your CTU currently adopt a consent process that specifically requests consent for patient's data to be used outside the
scope of the individual clinical trial?
i.e. Is it made clear to the patient that their data may be used for further secondary analysis in the future?
o	No o	Yes

6.	Would you be prepared to introduce and explain this clause to clinical trial subjects? [NOTE: Question #6 only asked of
participants who answered “No” to Question #5]

Data Sharing Requests – Experiences

The following questions allow you to provide details of occasions when your Clinical Trial Unit has been approached with a request for access to data you have generated at your unit or are a custodian of.

7.	How many requests to share data from trials your CTU has been involved in have you received in the last 12 months?
o	None o	1
o	2
o	3
o	4
o	5+


Experiences Of Being Approached for Data [NOTE; Questions #8 - #19 were not asked of participants who had answered “None” to
Question #7]

Please use the questions below to give an indication of the considerations affecting the outcome of data sharing requests.

8.	Who has approached your CTU with a request to access data?
Please indicate all applicable answers. o	NHS Trust/Clinician
o	Other UK CRC registered CTUs
o	Industry e.g. pharmaceutical company representatives o	Institutes of higher education e.g. universities
o	Independent researcher o	Other, please specify:

9.	What reason/s were given by the requester to justify the request for data sharing?
If the requestor offered multiple reasons please indicate all of them.
o	Developing/Evaluating novel statistical methods o	Meta-analysis
o	Developing study methods o	Teaching
o	Aiding design of future clinical trials o	Testing secondary hypotheses
o	Other, please specify:

10.    Were any requests for data fulfilled?
'Yes' indicates that data were shared with the requester.
o	No o	Yes


11.    Please indicate below if the request/s were fulfilled completely (as per request) or partially? (either enter "C" or "P") [NOTE;
Question #11 only asked of participants who answered “Yes” to Question #10]

12.    When assessing whether you were prepared to fulfil the request, were any of the following considered?
	Yes	No	Not applicable	
Timing of request
Historical sharing experiences	o
o	o
o	o
o	
Verification of governance
Labour intensity of fulfilling request
Patient consent	o
o
o	o
o
o	o
o
o	
Requester (i.e. only share with certain groups)
Pre-existing third party agreements	o
o	o
o	o
o	
Quality and originality of research proposal	o	o	o	

13.    Further to the considerations listed above, please detail any other issues that were or should have been raised when considering requests:

14.    Did you have any problems transferring the data in the format required by the requester?
o	No o	Yes


15.    Please indicate what the problems were? [NOTE; Question #15 only asked of participants who answered “Yes” to Question #14]

16.    To assess the request for data, were you provided with any of the following:
Please indicate any further information/documentation provided to validate the request.
o	Research proposal outlining the purpose of the data o	Data Sharing Agreement
o	Requester credentials /  competence to analyse
o	Assurances there would be no attempt to retrospectively identify patients
o	Other, please specify:

17.    In terms of labour and resources required can you compare whether the activities listed below were more difficult for historical data (>5 years old) compared to more recently generated data (within the last 5 years)?

Less difficult	About the same
More difficult	Not Applicable

Reviewing of request for validity	o	o	o	o
Location/Preparation of data into compatible
(shareable) format	o	o	o	o
Anonymisation of the data	o	o	o	o
Creating a Data Sharing Agreement	o	o	o	o
Other	o	o	o	o


18.    If you indicated 'other' in the question above, please indicate what other activities you refer to:

19.    Based on your previous experience, can you rate the below activities in terms of their labour implications and resource intensity?
Please define 'resource intensive' as a task that takes over 4 hours, i.e., half a day.

Likely to be resource intensive
Not likely to be resource intensive
Not applicable

Reviewing of request for validity	o	o	o
Location/Preparation of data into compatible (shareable)
format	o	o	o
Anonymisation of the data	o	o	o
Creating a Data Sharing Agreement	o	o	o


Experiences of Requesting Data

Please provide details of occasions when your clinical trial unit (or individual member thereof) has approached an external data custodian with a request to access data.

20.    How many requests have been made in the last 12 months?
Please include any data requests you are aware of from within your unit, including those requesting data for meta-analysis.
o	None o	1
o	2
o	3
o	4
o	5+


Experiences of Requesting Data (2) [NOTE; Questions #21 - #27 were not asked of participants who answered “None” to Question #20] Please use these questions to indicate how successful/unsuccessful experiences with data sharing requests have been.
21.    Please indicate who the requests for data were made to:
Please indicate all applicable answers.
o	NHS Trust/Clinician
o	Other UK CRC registered CTUs
o	Industry e.g. pharmaceutical company representatives
o	Institutes of higher education e.g. universities o	Independent researcher
o	Other, please specify:

22.    Were any requests for data fulfilled?
'Yes' indicates that data were received.
o	No o	Yes

23.    If your request was denied, please indicate the reasons why:
Please detail any reasons that were given by the data custodian for denying the request.

24.    Please indicate if your request was fulfilled:
o	Completely (as per request)
o	Partially (not all requested data were provided)

25.    If your request was fulfilled please indicate the length of time taken to receive the data from the first request:

26.    Did you have to provide any of the following to the data owner:
Please indicate any further information/documentation you had to provide to validate your request.
o	Research proposal outlining the purpose of the data o	Data Sharing Agreement
o	Requester credentials /  competence to analyse
o	Assurances there would be no attempt to retrospectively identify patients
o	Other, please specify:

27.    Please indicate the reasons for your request to access data:
i.e. meta-analysis

Future Perspectives

Please use these questions to indicate your views on the future of sharing of data within clinical trials.

28.	Please rate your knowledge of the following:	
Unaware	
Awareness	
Reasonable	
Good	
Excellent	
	
EMA Policy/0070	
o	only
o	
o	
o	
o	
	AllTrials Campaign
Yale Open Data Access (YODA) Project	o	o	o	o	o	
	GlaxoSmithKline's
www.clinicalstudydatarequest.com	o	o	o	o	o	
	BMJ Policy on Data Sharing
Clinical trial funder's policy on data sharing	o o	o o	o o	o o	o o	

29.    Further to the iniatives and policies mentioned above, are you aware of any other initiatives/data sharing policy?
o	No o	Yes

30.    If yes, please briefly describe the initiative(s) below:  [NOTE; Question #30 only asked of participants who answered “Yes” to
Question #29]

31.    In principle, would you be willing to transfer data to a centralised UK repository in the future (on the condition the repository is considered legal and ethical)?
i.e. within the next 2-3 years. Examples of shared platforms include  www.data-archive.ac.uk and  www.clinicaltrials.gov
o	No o	Yes

32.    Please provide a brief explanation for your answer above:
If your answer was 'no' please explain what assurances you would require to encourage you to say 'yes'.

Future Perspectives – Requirements

This line of questioning aims to establish the requirements for a data sharing platform, with the intention of developing guidelines to streamline the process.

33.    Do you have standard formats for your electronic data currently?
e.g. SNOMED CT, MedDRA, CDISC
o	No o	Yes

34.    Are there any specific reasons/external influences that would prevent your unit applying a standard data sharing policy?
'Standard' implies a data sharing policy that will be followed by all the UK CRC Registered CTUs (as a minimum)
o	No o	Yes

35.    If yes, please provide details: [NOTE; Question #35 only asked of participants who answered “Yes” to Question #34]

Future Perspectives – Models

If you were required to both request data from outside your CTU and also required to provide access to your CTU's individual patient data, please indicate which of the models below would you expect to be most suitable for your needs with regards to:
1) Data (anonymised individual patient data and associated trial documentation) transfer and storage
2) Approval process for data access
3) Timing of data access (i.e., when should data be shared?)

36.    Data (anonymised individual patient data and associated trial documentation) transfer and storage models:
−	Open access (data is uploaded to, and downloaded from, a central independent repository)
−	Access through interface (data remains with the data custodian but access is granted for external users to analyse data through a specific interface (such as that adopted by  www.clinicalstudydatarequest.com)
−	Transfer of data to external user (data transferred to external users for secondary analyses)
Please rate according to a scale based on suitability for the specific needs of the CTU you are affiliated to and only use each response once.

Most suitable	Not quite suitable
Least suitable

Open access                                                                                                             o                             o                            o Access through interface                                                                                       o                             o                            o Transfer to external user                                                                                       o                             o                            o


37.    Please briefly outline the main reasons for your response to the previous question:

38.    If there is an option that would be more suitable for your unit, please provide it below:

39.    Approval process for data access models:

−	Open access (no approval required, data available for any user to access)
−	Reviewed data access (specific detailed requests are placed with the custodian who assesses the request based on science, benefit-risk analysis, and competence of the requestor to perform the specified analyses)
−	Learned intermediary (an Independent Review Board reviews requests and judges them based on criteria such as science, benefit-risk analysis, and competence of the requestor to perform the specified analyses)
Please rate according to a scale based on suitability for the specific needs of the CTU you are affiliated to and only use each
response once.

Most suitable	Not quite suitable
Least suitable

Open access	o	o	o
Reviewed data access	o	o	o
Learned intermediary	o	o	o

40.    Please briefly outline the main reasons for your response to the previous question:

41.    If there is an option that would be more suitable for your unit, please provide it below:

42.    Timing for data access (i.e. when should data be shared?)
Please indicate which timeline you think is most suitable.
o	As soon as the trial is closed, data has been cleaned and final analysis has been completed o	Within 12 months after the end of the trial (defined as last patient last visit)
o	Within 24 months after the end of the trial (defined as last patient last visit)
o	At any time after the trial team have completed all analyses and secondary exploratory analyses
o	Other, please specify:


Future Perspectives – Potential Problems

There have been a number of issues highlighted that may arise from the sharing of complete clinical trial data. Please use this page to indicate your concerns and describe any problems you foresee.

43.    If there was a platform where full clinical trial data were made available for sharing, how concerned would you be about the following:

N/A		Not at all concerned
Not very concerned
Moderately concerned
Very concerned

Loss of IP/ability to publish                                                              o                     o                         o                         o                         o
Incorrect secondary analysis or misuse of data                           o                     o                         o                         o                         o Identification of patients                                                                  o                     o                         o                         o                         o Resource requirements to ensure data were uploaded
correctly (including historical data)                                                o                     o                         o                         o                         o
Gaining consent from patients for research using their
data outside of the specific trial they enrolled in                        o                     o                         o                         o                         o
Other                                                                                                    o                     o                         o                         o                         o

44.    If you indicated that you would be concerned about 'other' issues in the previous question, please briefly describe them below:

45.    Please indicate any problems you foresee if there was a call for the publication of all clinical trial data (including Individual
Patient Data) via a controlled access platform. Where possible, please explain what could be done to address these problems.

46.    Do you have any other perspectives/comments? Please provide details:

47.    Please indicate if you are happy to be contacted further about the issues of clinical trial data sharing:
o	No
o	Yes

Thank you for taking the survey!
